# Lieut. Guy Harle Irvine

**Published:** 1900-06

**Authors:** 


Lieut. Guy Harle Irvine, R.A.M.C., who was killed in the disaster at Sanna's Post, South Africa, on March 31st, was formerly a student at the University College and Royal Infirmary, Bristol.

He was educated at Wellington College. He commenced his medical studies in October, 1893, and qualified in October, 1898. He entered the R.A.M.C. in February, 1899, passing in tenth on the list. In the examination at the end of his course at Netley he obtained the second place, being first in four subjects.

Lieut. Irvine left England in October last, and was appointed to General French's Cavalry Brigade. At the time of his death he had medical charge of P, Q and U Batteries of the Royal Horse Artillery, and we hear that on the day of his death he was under fire for two hours dressing the wounded. His loss is felt very keenly at the University College and at the Royal Infirmary, where he was much esteemed by all with whom he was associated.

